# An attempt to evaluate the use of mixed reality in surgically treated pediatric oncology patients

**DOI:** 10.1038/s41746-025-01638-7

**Published:** 2025-05-09

**Authors:** Krzysztof Bronowicki, Justyna Antoniuk-Majchrzak, Iwona Malesza, Wiktor Możarowski, Agnieszka Szymborska, Bartosz Pachuta, Tomasz Walenta, Wojciech Jasica, Maciej Stanuch, Andrzej Skalski, Anna Raciborska

**Affiliations:** 1 Department of Oncology and Surgical Oncology for Children and Youth, Institute of Mother and Child, Warsaw, Poland; 2 Department of Artificial Intelligence and Innovation Medical Technology, Institute of Mother and Child, Warsaw, Poland; 3 MedApp S.A., Krakow, Poland; 4 Department of Measurement and Electronics, AGH University of Krakow, Krakow, Poland

**Keywords:** Cancer, Cancer imaging, Paediatric cancer, Sarcoma

## Abstract

Mixed reality (MR) technology is increasingly used in surgical procedures, particularly in pediatric oncological surgery. The CarnaLife Holo system (MedApp S.A., Poland) converts medical imaging data into interactive 3D holograms for preoperative planning and intraoperative use. This study presents a preliminary evaluation of MR’s impact on surgical procedure (SP) duration and hospitalization (H) time. A retrospective analysis of patients treated between 2023 and 2024 compared outcomes of surgeries performed with (*n* = 9) and without MR. Diagnoses included pulmonary metastases, sacrococcygeal tumor, clavicle tumor, aneurysmal bone cyst, soft tissue tumors, femoral and chest wall tumors. SP duration in the MR group was generally comparable to conventional methods, with hospitalization times remaining within typical ranges. Although a slight increase in procedure time was observed in a few cases, MR did not significantly prolong SP or H. MR appears to be a promising tool in pediatric oncological surgery. Further research on larger cohorts is warranted.

## Introduction

The development of modern medical technologies, ranging from virtual reality (VR) and augmented reality (AR) to mixed reality (MR), is significantly transforming the planning and execution of many surgical procedures. Collectively referred to as extended reality (XR), these technologies create new opportunities for diagnostic and therapeutic support, particularly in surgical oncology. VR is a technology that generates a fully immersive digital environment, isolating the user from the real world, while AR overlays digital elements onto the physical world, enabling interaction with both real and virtual components. In contrast, MR not only overlays digital content but also allows real-time interaction with three-dimensional (3D) holograms anchored in the real environment. This capability makes MR particularly useful for surgical navigation and intraoperative assistance, as it provides enhanced depth perception and spatial awareness. Several studies have explored the applications of XR technologies in surgery. While VR is primarily used for surgical simulation and preoperative planning, AR has demonstrated potential in intraoperative guidance by superimposing critical anatomical structures onto the surgical field. MR, however, offers a more advanced integration of digital models with real-world anatomy, allowing surgeons to interact with patient-specific 3D reconstructions in real time^1–10^.

MR is a new technology that blends the physical and digital worlds using the Head-Mounted Display (HMD). It enables a stereoscopic view of the images giving a sense of true 3D perspective on the visualizations. The holograms can be manipulated through hand gestures, voice commands, virtual menus, and eye tracking, allowing the operator to adjust the visualization without breaking the sterile field. Recent research has highlighted the benefits of MR in improving surgical precision, reducing intraoperative errors, and enhancing anatomical visualization, particularly in complex oncological cases^11–13^.

Existing reports regarding the use of MR in surgical oncology are primarily based on single cases or small groups of patients. Especially pediatric patients are underrepresented in the literature. Additionally, assessing the effectiveness of this technology using objective metrics remains challenging.

This study aimed to evaluate the potential of MR technology in pediatric oncological surgery. We focused on assessing the impact of MR on operative duration, and hospital stay length. Our findings were compared with existing literature to better understand the potential benefits of MR in pediatric surgical oncology.

## Results

MR was used in 9 pts. The general characteristics of patients from the control group and study group are presented in Table 1 and Table 2.

**Table 1:** Basic characteristics of the control group and the mixed reality group

	Control group					Mixed reality group			
Type of procedure^a^	Number of patients	Age – mean ± SD	Age - median	Gender		Number of patients	Age	Gender	Pathological evaluation
1	34	15.33 ± 4.45	15.95	F	11 (32.35%)	2	14.8, 9.2	F, F	Osteosarcoma, Rhabdomyosarcoma
				M	23 (67.65%)				
2	37	11.58 ± 5.43	12.18	F	12 (32.43%)	1	14.68	M	Osteoid osteoma
				M	25 (67.57%)				
3	34	11.28 ± 4.45	11.38	F	20 (58.82%)	1	11.47	F	Osteochondroma
				M	14 (41.18%)				
4	32	13.49 ± 4.18	13.82	F	14 (43.75%)	1	4.79	M	Immature teratoma G1
				M	18 (56.25%)				
5	28	13.10 ± 4.44	13.92	F	10 (35.71%)	1	13.22	F	Aneurysmatic bone cyst
				M	18 (64.29)				
6	33	11.88 ± 4.64	13.26	F	15 (45.45%)	2	18.74, 2.55	M, F	RMS alveolare, Lipoma
				M	18 (54.55%)				
7	17	12.46 ± 5.28	13.07	F	8 (47.06%)	1	9.04	F	Osteosarcoma
				M	9 (52.94%)				

^a^1 - thoracotomy of pulmonary metastases, 2 - biopsy of tumor of the left femur, 3 - resection of tumor of the left clavicle, 4 - resection of sacrococcygeal tumor, 5 - curettage and bone graft of aneurysmal cyst of the right femur, 6 - biopsy of soft tissue tumors of the lower limb-thigh, 7 - resection of a chest tumor.

**Table 2:** Duration of surgical procedures and hospitalizations in the control group and the mixed reality group

		Control group				Mixed reality group		
Type of procedure^a^	Number of patients	Median operation time [min]	Range [min]	Median hospitalization time [day]	Range [day]	Number of patients	Operation time [min]	Hospitalization time [day]
1	34	122	60–240	4	4–23	2	150, 125	13, 8
2	37	35	15–75	1	1–10	1	70	1
3	34	45	15–315	1	0–15	1	105	4
4	32	60	10–345	1	1–30	1	150	8
5	28	77	40–150	3	1–7	1	65	4
6	33	35	10–130	1	0–8	2	60, 40	1, 2
7	17	180	60–240	11	6–26	1	195	15

^a^- 1 - thoracotomy of pulmonary metastases, 2 - biopsy of tumor of the left femur, 3 - resection of tumor of the left clavicle, 4 - resection of sacrococcygeal tumor, 5 - curettage and bone graft of aneurysmal cyst of the right femur, 6 - biopsy of soft tissue tumors of the lower limb-thigh, 7 - resection of a chest tumor.

**Fig. 1:**
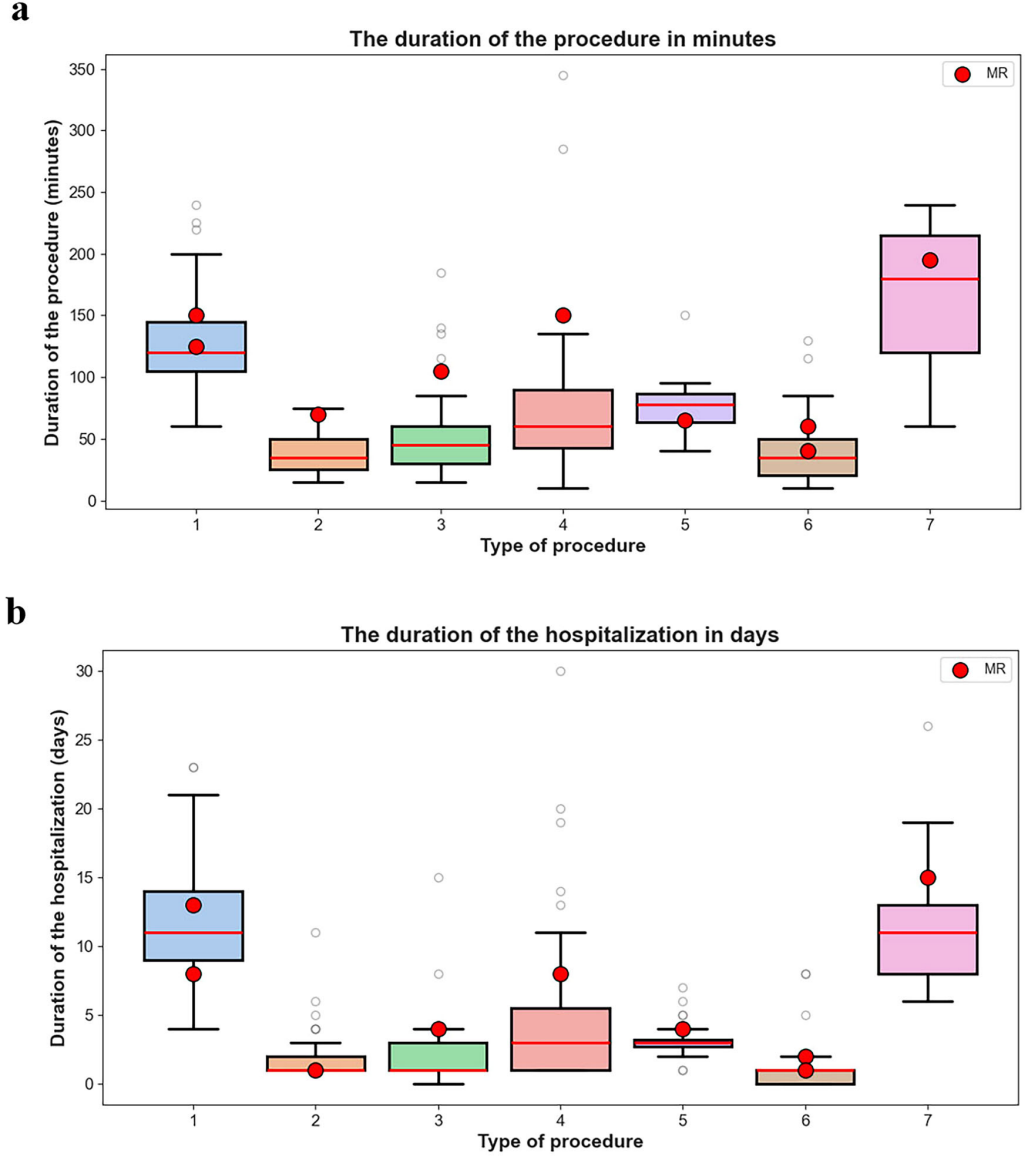
Comparison of surgical outcomes between the mixed reality (MR) group and the control group. **a** Average duration of operation (in minutes) for different procedures. (**b**) Average hospitalization time (in days) for the same procedures. Blue bars represent the control group, while orange bars represent the MR group. The procedures include: (**1**) thoracotomy of pulmonary metastases, (**2**) biopsy of a tumor of the left femur, (**3**) resection of a tumor of the left clavicle, (**4**) resection of a sacrococcygeal tumor, (**5**) curettage and bone grafting of an aneurysmal cyst of the right femur, (**6**) biopsy of soft tissue tumors of the lower limb (thigh), (**7**) chest tumor resection.

The procedure time for MR patients generally fell within the typical range observed in the control group, including the minimum and maximum values, further supporting their consistency with standard procedure durations. However, for procedures 3 and 4, the durations were slightly longer and extended beyond the whiskers of the boxplot. Nonetheless, given the small sample size (*n* = 1 for each procedure) and the fact that hospitalization durations remained within the expected range, these cases may not represent true outliers (Fig. 1).

## Discussion

The integration of XR technologies, including VR, AR and MR, into surgical procedures has significantly advanced three-dimensional imaging techniques. MR combines digital overlays with real-world visualization, allowing for real-time interaction between physical and virtual elements.

The number of publications on the use of MR in pediatric patients is limited, with most studies focusing on adults. MR has been mainly used in cardiac surgery, neurosurgery, maxillofacial surgery, urology, orthopedics, and surgical oncology including abdominal surgery. MR remains underrepresented in pediatric surgical oncology^1,11,12,14–29^. The use of MR in pediatric oncological surgery is particularly noteworthy and warrants further exploration.

In this study, MR technology was utilized in various pediatric oncological procedures, including: thoracotomy and resection of pulmonary metastases, biopsy of tumor of the femur, resection of tumor of the clavicle, resection of sacrococcygeal tumor, curettage and bone graft of aneurysmal cyst of the femur, biopsy of soft tissue tumors of the lower limb-thigh and resection of the chest tumor.

The literature has repeatedly highlighted the benefits of MR in both adult and pediatric patients, including improved visualization, enhances surgical navigation accuracy, and may reduce operative times. Many authors support also the opinion that MR provides new possibilities in cases where conventional imaging techniques fail to provide sufficient localization of critical structures^2–7,9,10^.

**Fig. 2:**
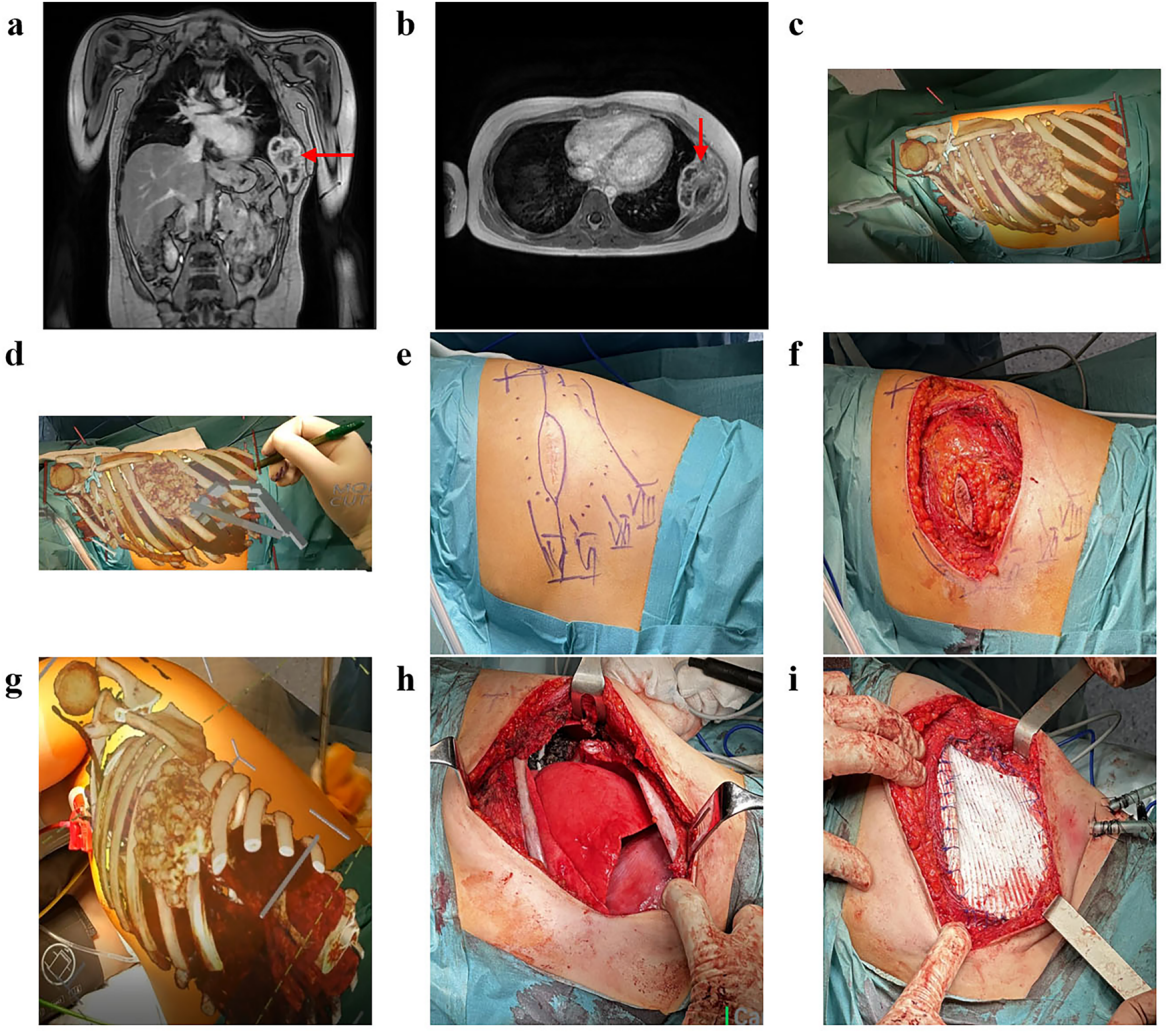
Individual stages of applying and interacting with the hologram. **a**–**e** represent the preoperative phase and (**f**–**i**) illustrate intraoperative navigation and tumor resection. The red arrows in a and b indicate the tumor.

This study was based on cases suspected of oncological conditions. The 3D holograms were generated from preoperative imaging and evaluated both before and during surgery. The holograms were overlaid onto the patient’s body and allowed interaction via hand gestures, voice commands, and virtual menus. Initially, the hologram was overlayed by placing selected anatomical points followed by radiological markers placed on the patient in the locations corresponding to those used during the imaging examination. Our experience led to development of a structured workflow consisting of preoperative planning with the hologram, application of the hologram onto the patient’s body before surgery, and tumor resection with periodic reference to the hologram during the procedure (Fig. 2).

It seems, that in the pediatric population, precise surgical planning is crucial, as the preservation of healthy tissues and anatomical structures is essential for the child’s ongoing development.

Similar conclusions have been drawn in other studies. For instance, Wellens demonstrated that 3D kidney models improved anatomical understanding among the surgeons and were helpful for preoperative planning of nephron-sparing surgery (NSS) for Wilms tumors^2^. Similarly, Chaussy stated that 3D representations allow surgeons to better anticipate the operative risks and can also serve as an additional tool for better selecting patients for NSS^4^.

The benefits of MR for surgical planning have also been highlighted in pediatric cardiac surgery. Gehrsitz and Brun emphasized its role in preoperative planning^3,6^. Regarding the assessment of precision during the procedure, Tsai demonstrated that there was no significant difference between the total coronary bypass graft length on hologram and computer tomography (CT). In his study, the hologram and CT measurements were highly correlated, with an excellent agreement^30^. Whereas Cho stated that the probability of a surgeon achieving a 10 mm surgical margin with a 3 mm tolerance was 90.2% in AR-assisted resections compared to 70.7% in conventional resections^31^.

3D visualization methods, including MR and 3D printing, have demonstrated superiority over conventional DICOM imaging. Lau, in a comparative study of both MR and 3D-printed models, noticed that both were superior to standard DICOM images in the visualization and management of congenital heart diseases^32^. Additionally, the quality of DICOM images plays a crucial role in the reconstruction of 3D models^1,2,8,33^.

Likewise, Fu reported that MR can be a feasible approach for localizing pulmonary nodules. In his study, 94% of nodules were accurately localized using MR, compared to 30% identified through manual palpation^34^.

Based on this data, Rahul suggested that greater emphasis should be placed on the use of MR for surgical navigation^35^. Also, Tao observed that the application of MR to sentinel lymph node biopsy (SLNB) in breast cancer can significantly reduce the detection time and complication rates, while also improving patient satisfaction^36^. Pelanis supported this view, demonstrating in his study the use of 3D liver models in MR significantly decreased the median time for tasks requiring a spatial understanding of the organ from 23.5 s using magnetic resonance imaging (MRI) images to 6.0 s using HoloLens^9^.

Likewise, Gehrsitz demonstrated that the use of 3D holograms reduced the intraoperative preparation time and allowed a previously unattained level of comprehension of anatomy and pathology in preoperative planning^3^. Wierzbicki maintains that the combination of minimally invasive surgical techniques with CarnaLife Holo resulted in a 1/3 reduction in procedure time compared to surgeries performed without the CarnaLife Holo system^5^.

These observations, which align with our findings, support the view that 3D visualization methods should be considered a valuable supplementary tool in preoperative planning and operative routine, as they do not significantly prolong operative time. However, it is important to account for the learning curve associated with MR technology, as the surgical team must adapt to the software and its functionalities. In our study, before each procedure involving CarnaLife Holo system, the surgical team received dedicated training on how to operate the system. With each subsequent procedure, interaction with the application has become more efficient, and the overall workflow improved progressively. As familiarity with the technology increased, surgeons were able to integrate MR into their practice with greater ease, minimizing potential disruptions to the surgical process. A well-executed procedure is generally associated with an uncomplicated surgical course^5,10,13^. Similarly, our results indicate that the use of AR and MR does not significantly affect hospital stay compared to traditional methods. The surgical teams involved in the study provided positive feedback regarding the use of MR technology. However, we did not conduct formal surgeon satisfaction surveys, as our focus was on evaluating other, potentially more objective factors related to the use of MR in our patients. All procedures were performed by experienced senior surgeons. The entire surgical team particularly appreciated the educational benefits of 3D visualization, emphasizing its value in anatomical orientation and intraoperative decision-making.

Another promising direction for MR development could be its application in more complex, multi-stage procedures, such as resections of large metastatic tumors involving critical anatomical structures. Research on integrating MR with imaging modalities like MRI and CT suggests that personalized holographic models could support surgeons in accurately planning these procedures^3,6,37^.

MR also has significant potential in surgical education and training. Realistic holographic models enable young surgeons to simulate complex clinical cases in a controlled environment, significantly enhancing their preparedness for real-world operating room scenarios^38–40^. Several studies on the use of this method in surgical education, such as the reports by Kaplan and Nguyen, confirm that this approach improves spatial understanding of anatomy and enhances procedural accuracy^41–43^. Similarly, Cho et al. noted that AR-based navigation improves surgical accuracy in both experienced and inexperienced hands^31^. Additionally, Borhar and Lu highlight the role of MR in patient education, emphasizing its potential to improve patient understanding and engagement in their treatment process^32,44^.

Additionally, standardizing MR-based procedures and integrating them with other surgical assistance technologies, such as robotics, indocyanine-green (ICG), or ultrasound systems, remains a challenge^8,45–48^.

AR and MR technologies offer extensive opportunities for further development and application in pediatric surgery, particularly in oncological cases. Future research could focus on integrating AR and MR with other advanced technologies, such as artificial intelligence (AI), which could assist in delineating tumor margins and predicting complications in real-time. Additionally, these systems could also dynamically adapt to anatomical changes during surgery, addressing one of the ongoing challenges in the practical application of XR^2,47^.

Despite its significant potential, MR faces several challenges that must be addressed to enable its widespread adoption in clinical practice. One of the major obstacle is the precise real-time registration of holograms with physical anatomy, particularly during procedures involving tissue deformation^3,8,10,49^. Therefore, developing technologies that integrate simulated lung deflation into holograms for pulmonary surgery appears to be a promising direction for future research^50^.

Some authors highlight ergonomic challenges associated with this technology, particularly the weight and design of HMD and their impact on head and neck musculature, as well as visual fatigue. Additionally, the field of view may be limited and the parallax effect can occur - a phenomenon in which background content moves at a different speed compared to content that is positioned in the foreground^51^.

Another technological limitation is the relatively short battery life of the HMD, which can pose difficulties during long surgical procedures^51–53^.

Economic aspect is another concern, as some authors emphasize the financial burden of MR technology^1,2,8,33^. Due to the non-profit implementation and pilot nature of the project, we did not estimate the costs of software or necessary hardware. Such costs should be taken into account in the future analyses. Although our study did not assess the financial aspects of MR implementation, it is important to consider its potential economic benefits. A reduction in operative time and shorter hospital stays may decrease the risk of postoperative complications, leading to more efficient resource utilization and overall cost savings for hospitals. By improving surgical workflows and procedural accuracy MR could enhance the cost-effectiveness of oncological treatment. Future studies should include a detailed economic analysis to evaluate the long-term financial impact of MR in clinical practice. Despite these challenges, the generally positive experiences reported with MR technology, and its preliminary benefits suggest that it holds promise for broader clinical implementation.

MR technology holds significant potential in pediatric oncological surgery. However, there is a lack of studies utilizing objective metrics and larger, homogenous groups of surgical procedures.

While most authors assess this technology positively, their evaluations are often subjective and based on varying evaluation scales^2,6,7,9,28,32,35,38,44,47,51^.

Despite promising results, this study has several limitations. The small sample size (*n* = 9) limits the statistical power of our findings and prevents definitive conclusions regarding the overall efficacy of MR in pediatric oncological surgery. Additionally, potential biases, such as selection bias and variability in surgical technique, may have influenced the results. Future research should prioritize prospective trials with larger, more homogenous patient cohorts to generate more robust evidence.

Our findings suggest that MR can be integrated into pediatric oncological surgery without prolonging operative times or hospital stays. Therefore, SP duration and H length may serve as important objective factors in assessing the usefulness of MR, especially in pediatric surgical oncology. Despite existing challenges, further studies, involving larger pediatric patient cohorts and diverse clinical subgroups are essential to fully evaluate the applicability of this technology in routine clinical practice. Additionally, cost analyses and long-term outcome studies will be essential in determining its feasibility for widespread adoption.

## Material and methods

### Introduction CarnaLife Holo

CarnaLife Holo (MedApp S.A., Poland) is an innovative solution that uses MR to change DICOMs and segmentations into interactive holograms that help with procedure planning and can be also used intraoperatively^5,54,55^. Based on the imaging data the user can create visualizations that are dedicated to different procedures and surgical sites thanks to predefined presets (transfer functions). The system allows to creation of segmentations of the pathology and surrounding tissues or blood vessels that are around the target. It is possible to overlay the created image in Real Size on the patient to get a clear view of the pathology and its localization in the patient. The system gives also the possibility to perform complex measurements in holographic space^30^.

### Technical aspects

The system consists of a high-performance workstation, Head-Mounted Display (HMD) - Microsoft HoloLens 2, and a router ensuring wireless communication between the HMD and the workstation. The input to the system can be medical imaging data in the DICOM format (CT, CBCT, MRI, ECHO, Ultrasound) or files containing models or segmentation results.

### Preoperative stage

The diagnostic imaging data are loaded into CarnaLife Holo as DICOM files (CT/MRI). The images are reconstructed in 3D and the user chooses the proper Preset and adjusts the histogram window to enhance the image and visualize all of the critical parts of the patients that are important from the surgical perspective. In case, the visualization is still not providing enough information, a segmentation can be performed to enhance the important structures and pathologies.

In case the patients’ overlay is needed, in the preoperative phase the user needs to mark the position of the anatomical landmarks or radiological markers in the data and save them in the saved state file for later use.

**Fig. 3:**
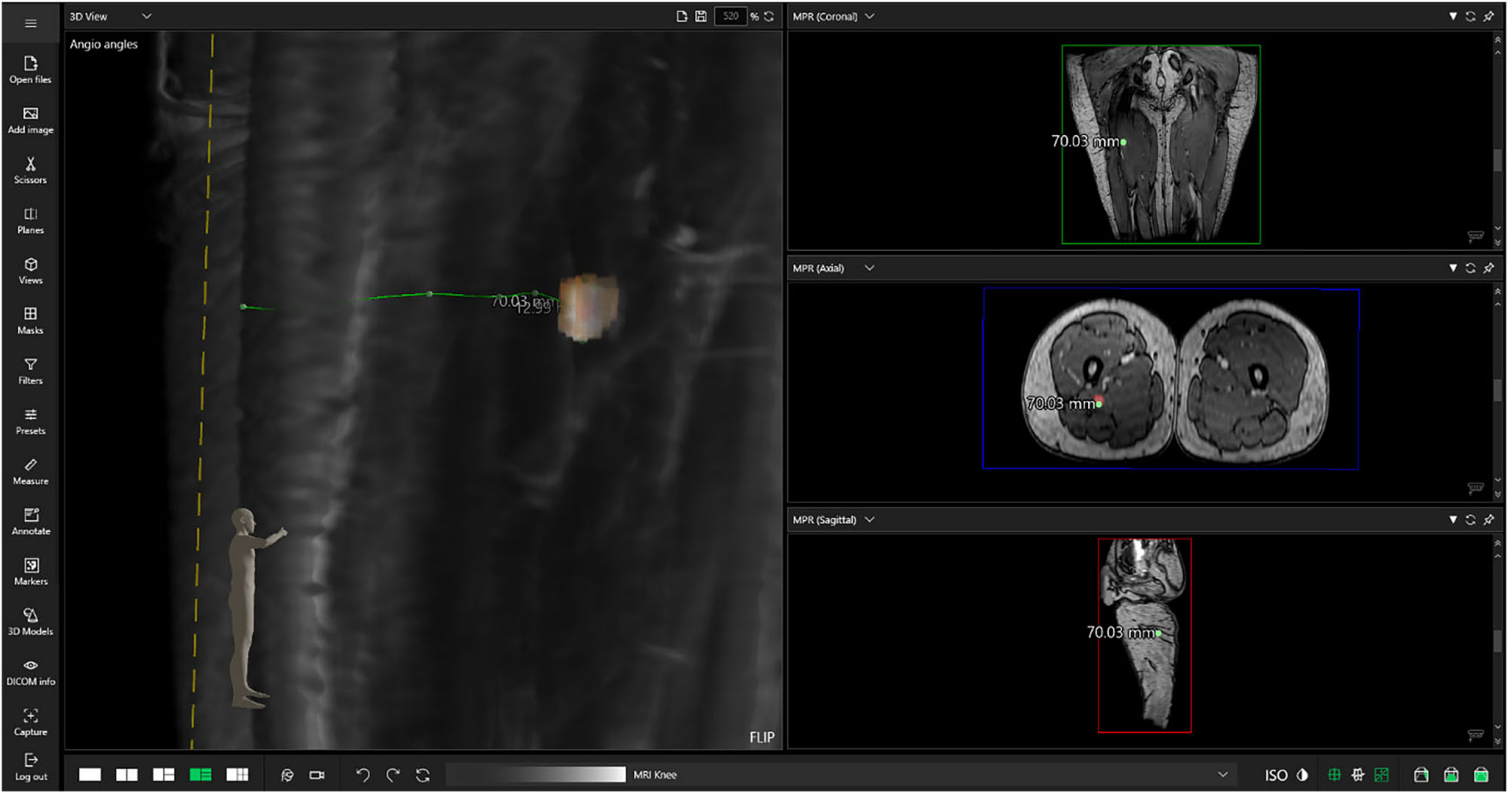
Preoperative radiological imaging is used for hologram generation. The diagnostic imaging data (Computed Tomography (CT) and Magnetic Resonance Imaging (MRI)) are processed as DICOM files, reconstructed in 3D, and adjusted to enhance visualization of critical anatomical structures. These images serve as the basis for holographic models used in surgical planning.

The system enables the user to create measurements based on 2D views and also from the HMD which provides much greater flexibility on the matter (Fig. 3).

### Intraoperative usage

The hologram can be overlayed by placing radiological markers on the patient in the areas that correspond to the markers placed during the imaging examination.

In the first step, the visualization state with the location of 4 markers needs to be loaded into CarnaLife Holo and then detected on the patient by using Hololens 2 glasses. After all the markers are found in Mixed Reality, the image is overlayed on the patient’s body using appropriate voice commands and hand gestures.

**Fig. 4:**
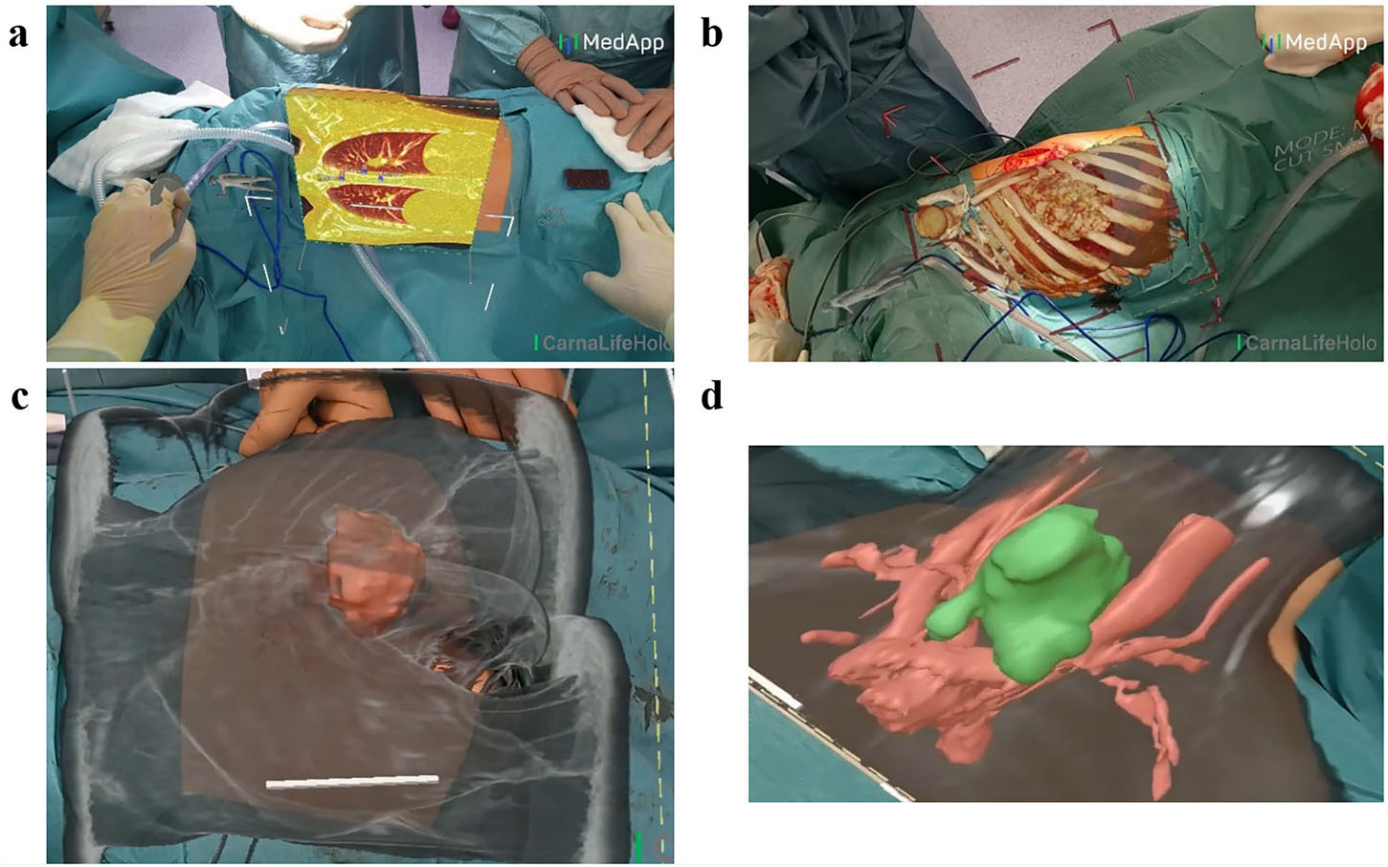
Intraoperative visualization using a hologram overlay. The holographic model, generated from preoperative Computed Tomography (CT) and Magnetic Resonance Imaging (MRI) data, is aligned with the patient's anatomy by detecting radiological markers and using voice commands and hand gestures. A printed reference point ensures stable positioning throughout the procedure, allowing realignment if the overlay is lost. The system enables real-time visualization of internal anatomical structures, assessment of critical elements such as major blood vessels, and intraoperative measurements without breaking the sterile field. The figure presents surgical procedures using MR-assisted intraoperative visualization: (**a**) thoracotomy, (**b**) resection of a chest tumor, (**c**) resection of a tumor of the left clavicle, and (**d**) resection of a sternal end clavicular tumor.

Next, in order to protect against loss of image positioning due to random factors, a printed reference point (a set of graphic codes) can be placed in the operating room. After the image overlay process, the reference point is scanned, and the state of the visualization is saved with information about the location of the hologram, the location of the reference point, and the spatial relationship connecting the above-mentioned objects. (Fig. 4)

During the procedure, if there is a loss of hologram overlay on the patient, it is possible to precisely reconstruct the position after scanning the reference point. Thanks to the lack of change in its position and having previously recorded information about the spatial relationship between the target position of the hologram and the reference point, it is possible to bring the overlay back on the patient without the need for recognition of the markers on the patient’s body.

Throughout the procedure, head movements can create a cutting plane, allowing internal anatomical structures and pathologies to be visualized from any angle and depth. This enables assessment of both the location of the pathology and critical structures, such as major blood vessels. Additionally, intra-procedural measurements can be performed without breaking the sterile field. For minimally invasive procedures, the access point and tool trajectory can be planned in advance.

### Study sample characteristics

A comparison was conducted between patients treated in 2023-2024 who underwent the same surgical procedures with or without the use of mixed reality system. The study include 6 female (66,7%) and 3 male (33,3%) patients, with an age ranged of 2.55 to 18.74 years (mean ± standard deviation: 10.94 ± 4.83 years, median: 11.47 years). The duration of surgical procedures (SP) and hospitalizations (H) were analyzed for all patients. No medical complications were observed in this group, and all surgeries were completely successfully.

The approval for this retrospective study was obtained by a multidisciplinary scientific Institutional Review Board of the Institute of Mother and Child, in accordance with applicable national legislation and international ethical standards for research involving Human participants, including the principles of the Declaration of Helsinki. The Institute of Mother and Child functions both as a research institute and a healthcare facility. As a research institute, its statutory activities permits the use of de-identified patient data for non-interventional scientific studies, including retrospective and prospective observational research that does not involve experimental treatment or additional medical procedures.

MedApp S.A., the developer of the CarnaLife Holo system, took part in the first edition of the Mother and Child Startup Challenge (MCSC), which was evaluated by a multidisciplinary scientific Institutional Review Board of the Institute of Mother and Child.

### Statistical analysis

Descriptive statistical methods were applied to summarize the data, including the calculation of mean, median, standard deviation, quartiles, range, skewness, and kurtosis. The results from patients who underwent procedures using MR were compared to those of the control group based on their distribution across quartiles and percentage differences relative to the median. Due to the small number of cases involving MR, the analysis was limited to descriptive characteristics without statistical significance testing. All statistical analyses were conducted using Python version 3.12.2 in Visual Code Studio version 1.97.0.

## Data Availability

Individual, de-identified participant study data will be shared upon reasonable request for the purpose of replication by contacting the corresponding author KB or JAM.

## Code availability

Individual, de-identified participant study data will be shared upon reasonable request for the purpose of replication by contacting the corresponding author KB or JAM.
